# Supplementary material for: Cellular Model of Malignant Transformation of Primary Human Astrocytes Induced by Deadhesion/Readhesion Cycles
Source: Int J Mol Sci. 2022 Apr 19;23(9):4471. doi: 10.3390/ijms23094471 (PMC9103552; doi:10.3390/ijms23094471)
Supplement: Supplementary file 1 [file ijms-23-04471-s001.zip › ijms-1513917-Supplementary Materials.pdf]

Supplementary Materials

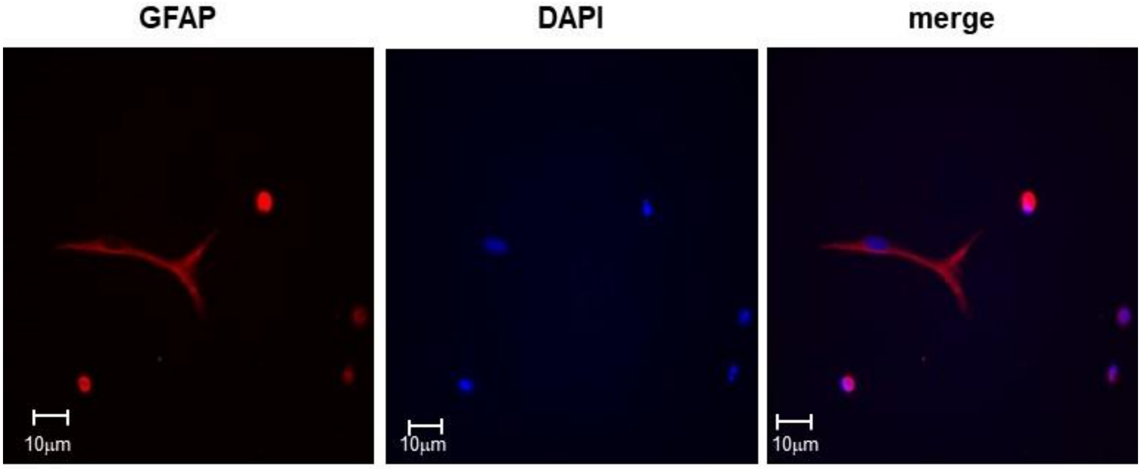

Figure S1. GFAP staining of primary human astrocytes. GFAP expression by primary human astrocytes. GFAP expression was evaluated in primary astrocytes by immunofluorescence using specifc antibody. Representative image of primary astrocytes stained with FITC-conjugated antiGFAP antibody (red) and DAPI-stained nuclei (blue). Magnification of 40x, scale bar of 10μm.

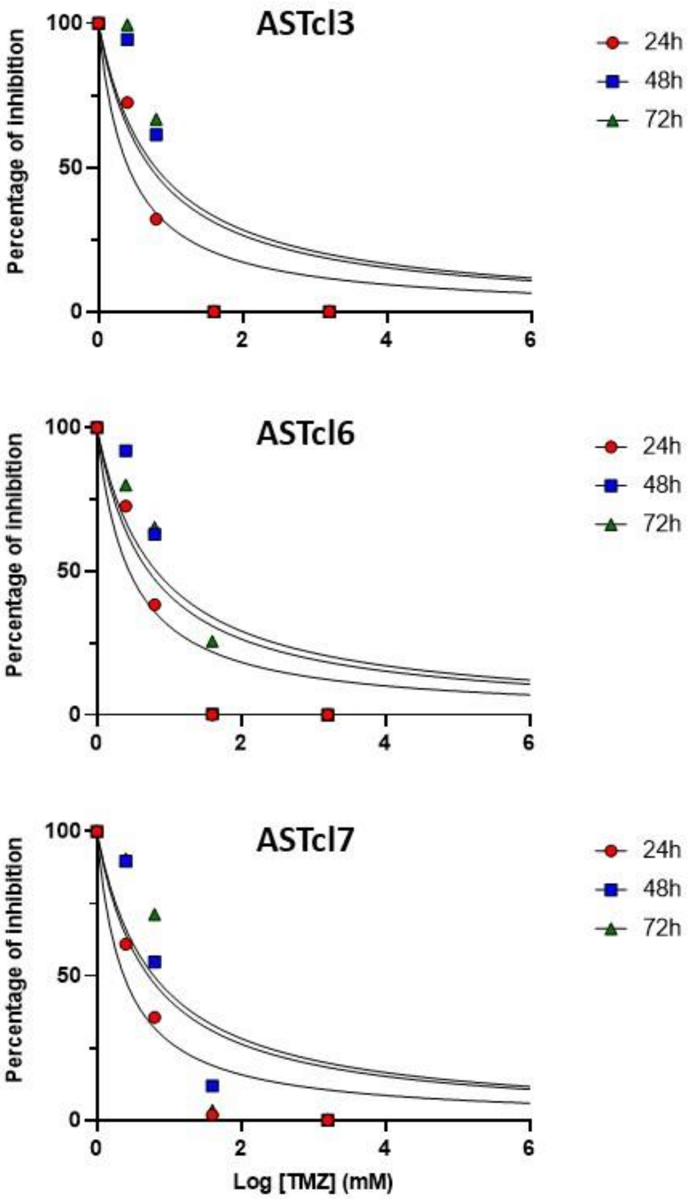

Figure S2. Cell viability of ASTcl3, ASTcl6 and ASTcl7 cells under TMZ treatment for 24, 48 and 72 hours. Data were analyzed by TMZ vs. normalized response (Log scale). IC50 was determined for ASTcl3 (0.7223 mM), ASTcl6 (0.7189 mM), and ASTcl7 (0.7175 mM) for treatment with TMZ for 48 hours. The concentration of 0.72 mM was used for all clones for assays that used treatment with TMZ.

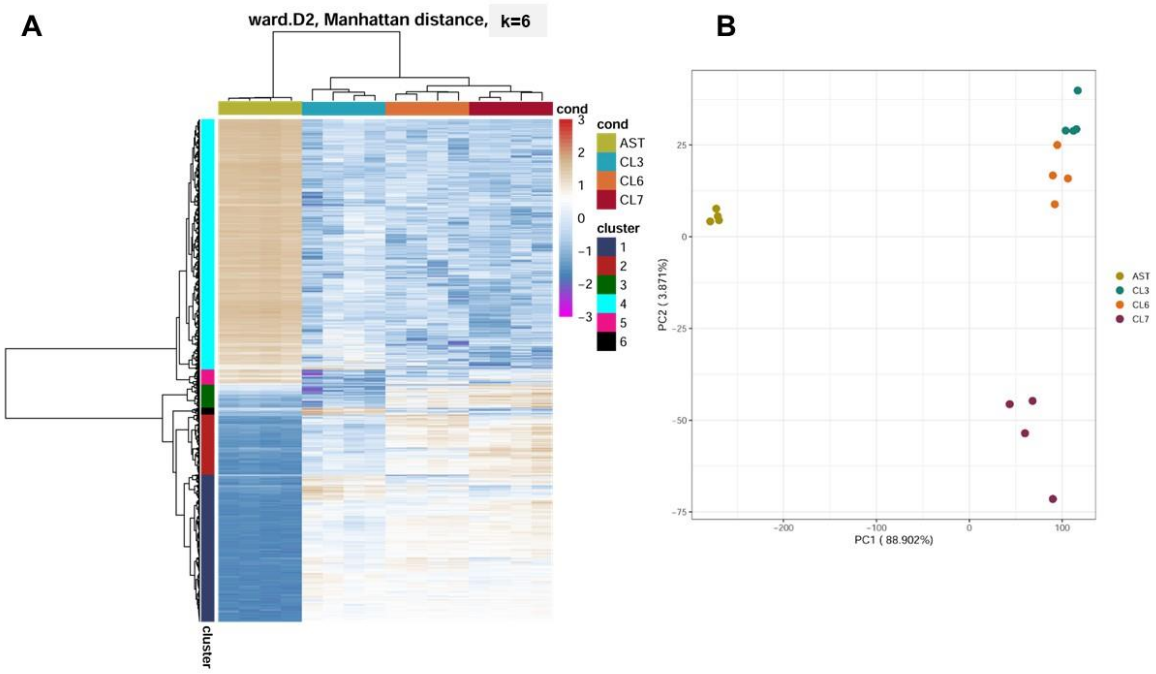

Figure S3. Astcl3, Astcl6 and Astcl7 have different transcription profile compared to primary astrocytes. **A.** Hierarchical clustering identifies 6 transcriptional modules (color-coded in the right side of panel) among differentially-expressed genes between the different astrocytes clones (CL3, CL6 and CL7) and the parental primary astrocytes (AST). Expression levels of genes (rows) are centered in the mean of each row and are color-coded by the gradient indicated in the right side of the heatmap. **B.** Principal component analysis (PCA) of the normalized RNAseq data. Differences are observed between primary astrocytes and derived clones and among clones, ASTcl7 being the most different.

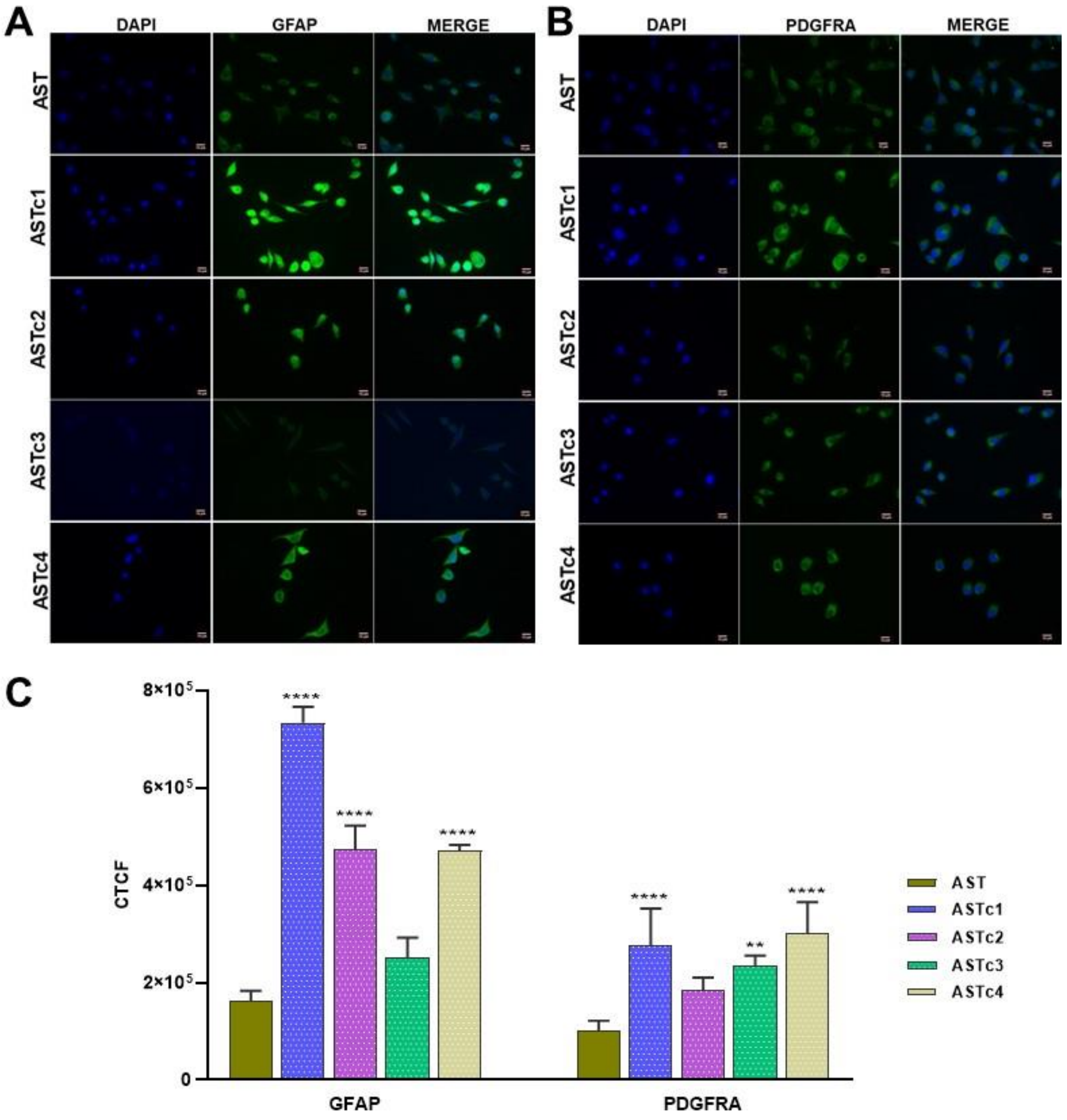

Figure S4. Astrocytes subjected to 1, 2, 3 or 4 deadhesion-readhesion cycles (Astc1, Astc2, Astc3 and Astc4, respectively) express high levels of GFAP and PDGFRA compared to primary astrocytes. A. Immunofluorescence staining for GFAP (green) and nucleus (DAPI-blue). B. Immunofluorescence staining for PDGFRA (green) and nucleus (DAPI-blue). C. Quantification of GFAP and PDGFRA staining. CTCF, corrected total cell fluorescence. Magnification of 40x, scale bar of 10µm. C. Statistically significant differences of each cell type compared to primary astrocytes are represented by asterisks: ** $p<0.01$ and **** $p<0.0001$.

	ASTctr		AST1c		AST4c		ASTcl3		ASTcl6		ASTcl7	
**Locus**	**Alelo 1**	**Alelo 2**	**Alelo 1**	**Alelo 2**	**Alelo 1**	**Alelo 2**	**Alelo 1**	**Alelo 2**	**Alelo 1**	**Alelo 2**	**Alelo 1**	**Alelo 2**
**D21S11**	28	32.2	29	30	28	32.2	28	32.2	28	32.2	28	32.2
**D5S818**	11	12	12	12	11	12	11	12	11	12	11	12
**D13S317**	8	11	10	10	8	11	8	11	8	11	8	11
**D13S317**	8	9	11	12	8	9	8	9	8	9	8	9
**D16S539**	12	12	9	11	12	12	12	12	12	12	12	12
**CSF1PO**	10	11	10	12	10	11	11	12	10	11	10	11
**Amel**	X	Y	X	Y	X	X (Y?)	X	X (Y?)	X	X (Y?)	X	X (Y?)
**vWA**	15	17	17	17	15	17	15	17	15	17	15	17
**TPOX**	8	8	8	8	8	8	8	8	8	8	8	8

Table S1. Cell lines established by deadhesion/readhesion cycles were proved to be derived from primary human astrocytes by genetic authentication. Allele detection at the loci in primary human astrocytes (ASTctr), first and fourth cycles of deadhesion/readhesion (AST1c, AST4c) and derived clones (ASTcl3, ASTcl6 and ASTcl7).
